# Retained Bullet in the Orbit Following a Gunshot Wound: Management of Subsequent Complications

**DOI:** 10.7759/cureus.108461

**Published:** 2026-05-07

**Authors:** Álvaro Bengoa-González, María D Lago-Llinás, Sara De-Matías-Gil, Patricia Brañas, Enrique Mencía-Gutiérrez, Consuelo Mata-Beltrán

**Affiliations:** 1 Department of Ophthalmology, 12 de Octubre Hospital, Complutense University, Madrid, ESP; 2 Department of Clinical Microbiology, 12 de Octubre Hospital, Complutense University, Madrid, ESP

**Keywords:** bullet, gunshot wound, orbit, pocket pistol, surgery

## Abstract

Gunshot wounds to the craniofacial region are often fatal, and isolated orbital involvement is exceptionally rare. Severe ocular and systemic complications, such as vision loss, neurological damage, and infections, can arise from high-velocity bullet injuries to the orbit. The case presented involves a male patient with two cranial impacts, one of which left a projectile lodged in the retrobulbar space. The initial treatment was surgical reparation of the wounds and observation. However, due to a persistent fever and the development of an abscess, the orbital projectile was removed. The patient’s condition improved, but amaurosis remained. In this instance, timely surgical removal of the projectile, guided by a customized risk-benefit analysis, early localization using computed tomography for accurate assessment, and multidisciplinary management to address complex injuries were all crucial elements. Orbital injuries caused by firearms are uncommon and extremely complicated, necessitating timely and individualized treatment to optimize the prognosis. Clinical evaluation should always be the basis for projectile removal decisions, weighing potential risks against benefits.

## Introduction

Gunshot wounds to the head and neck are often fatal, with only about one-third of victims surviving long enough to reach the hospital [1]. Isolated orbital gunshot wounds, in particular, are extremely rare, and their exact incidence remains unknown [2]. Adolescent male cases make up the majority of reported cases, which are usually connected to unintentional shootings or suicide attempts [3].

Because of their high morbidity and dismal prognosis, orbital bullet injuries are a type of high-velocity trauma that poses serious clinical challenges. These injuries frequently lead to both ocular and systemic complications [4]. The extent of damage depends on factors such as projectile size, velocity, and trajectory [5]. High-velocity bullets often cause extensive destruction of the orbit, adjacent paranasal sinuses, and brain [6]. Trauma to adjacent facial and intracranial structures often coexists with these injuries. This complexity demands a multidisciplinary approach and multiple surgical interventions for optimal management [7].

Ocular and neurological problems, such as vision loss, deficiencies in the central or peripheral nervous system, and infections, are the most frequent sequelae of these injuries. The decision to remove a projectile from the orbit should be made on a case-by-case basis, considering factors such as size, accessibility, risks, complications (including fractures or eye injuries), and legal considerations [8,9]. Despite advances in surgical techniques, the prognosis for severe orbital bullet injuries remains guarded. Early intervention and comprehensive management are critical to improving outcomes [4].

## Case presentation

**Figure 1:**
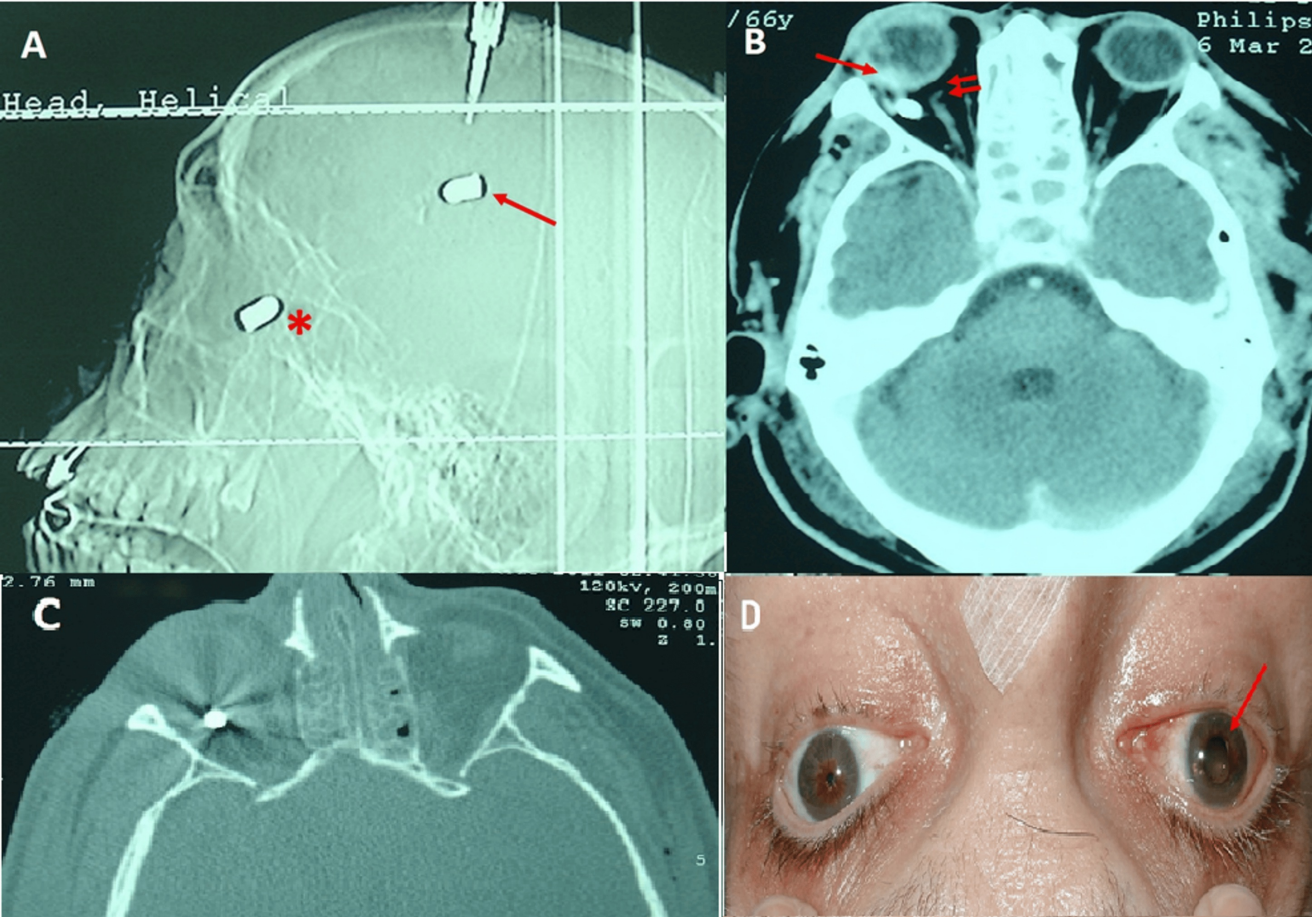
(A) Lateral skull X-ray showing two metallic projectiles: one in the cranial subgaleal zone (arrow) and one in the left orbit (asterisk). (B) A retrobulbar metallic bullet, retinal hemorrhage (arrow), proptosis of the eyeball, an intact left eyeball, and potential avulsion of the optic nerve (double arrow) are all visible on an axial CT image. (C) An orbital bullet with image distortion can be seen in an axial CT scan. (D) Proptosis and pupil mydriasis are visible in clinical photography of the right eye (arrow) CT: computed tomography

A 67-year-old male patient presented with two entry wounds in the left frontal region after suffering a severe traumatic brain injury from a firearm. Two retained projectiles were detected by computed tomography (CT) (Figure 1A). One entry wound was located on the left frontal side, with an anterosuperior horizontal trajectory relative to the lateral ventricles. A right frontal exit wound was associated with a 2 cm burst fracture. Along the bullet’s path, areas of parenchymal bleeding, gas, and bone fragments were observed. A subgaleal projectile was visible adjacent to the exit wound.

Another left frontal entry wound was situated anterosuperior to the first, with an oblique tract traversing the anterior frontal region. This trajectory crossed the frontal sinus and right superior orbital rim, involving the frontal and right maxillary sinuses, where bleeding was noted.

Additionally, a projectile was identified in the retrobulbar space (Figures 1B, 1C). There was an orbital floor fracture, but no loss of ocular sphericity or intraocular foreign body was found. A left frontotemporal craniectomy was performed as part of the surgical procedure to debride the wound, extract the cranial subgaleal projectile, remove bone fragments, and evacuate the hematoma. The orbital projectile was initially left under observation.

**Figure 2:**
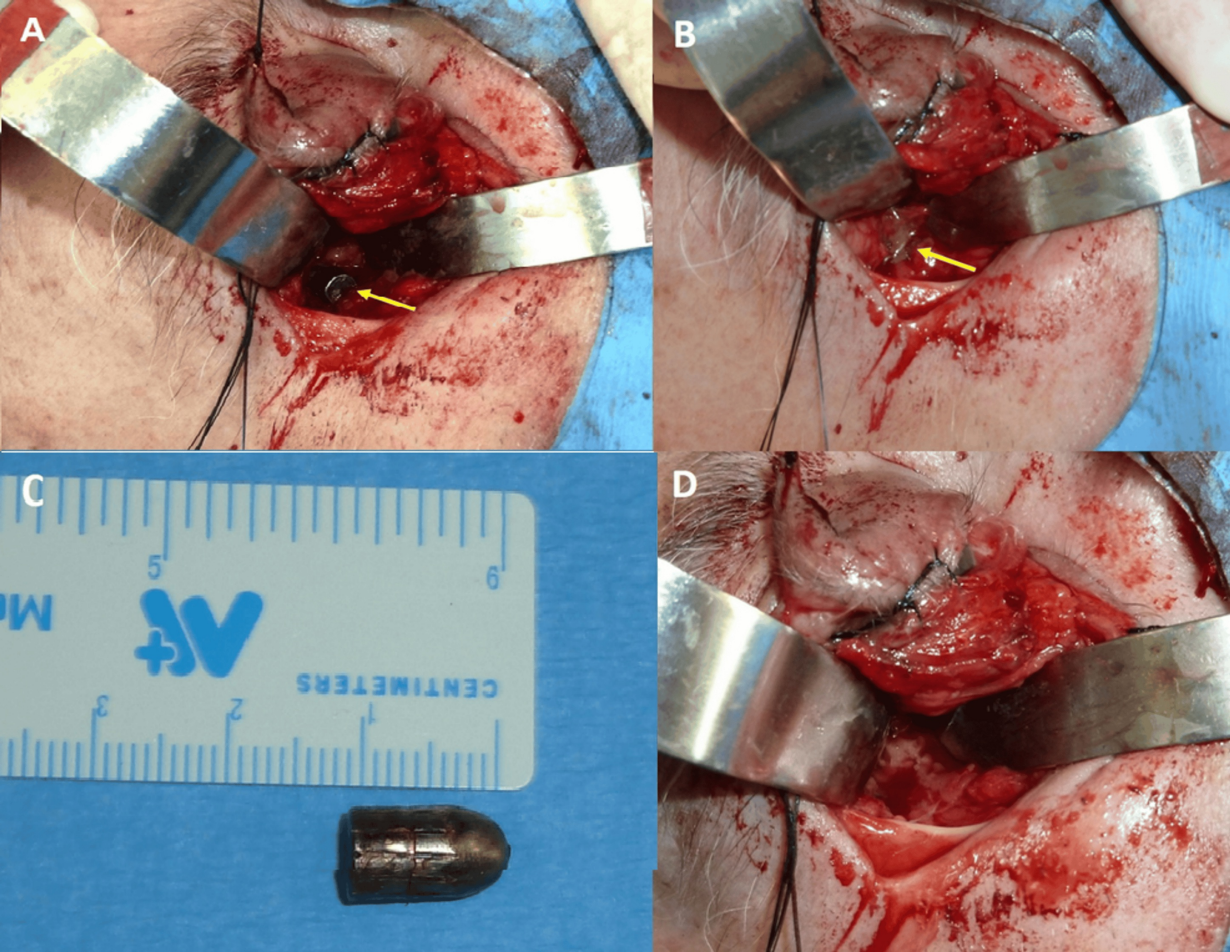
(A) The metallic bullet inside the left orbit is visible during the lateral orbitotomy procedure (arrow). (B) The surrounding inflammatory and purulent tissue is visible in the surgical field following bullet extraction (arrow). (C) The extracted bullet, with a diameter of 4.25 mm and a length of 12 mm. (D) After complete debridement, a clean surgical field guarantees that all inflammatory and purulent tissue has been removed

The initial ophthalmological examination was carried out after clinical stabilization. However, visual assessment was not possible due to the patient's comatose state. Clinical findings at this stage included mild proptosis, preretinal hemorrhage, and nonreactive mydriasis, without evidence of eyeball laceration (Figure 1D). Despite prophylactic antibiotic therapy from the outset, the patient develops persistent fever, prompting extraction of the projectile. A canthotomy was performed using an inferior transconjunctival approach, extended to a lateral orbitotomy. During the procedure, the bullet and an adjacent abscess were identified and removed. Samples were taken for culture, the area was irrigated with antibiotics, and a titanium mesh was placed to repair the orbital fracture (Figure 2). The antibiotic used as prophylactic treatment was amoxycillin/clavulanic acid due to possible bronchial aspiration. On the fifth day after orotracheal intubation, it was changed to ceftriaxone and metronidazole. And on the seventh day, given the possibility of an orbital abscess due to the development of high fever and leukocytosis, treatment with meropenem 2 g iv/eight hours and linezolid 600 mg iv/12 hours was started. The microbiological study confirmed the presence of *Staphylococcus epidermidis,* with an antibiogram showing sensitivity to the latter antibiotics, so they were maintained for five more days, with improvement in clinical condition. The antibiotic used to irrigate the abscess area during the surgical procedure was gentamicin.

**Figure 3:**
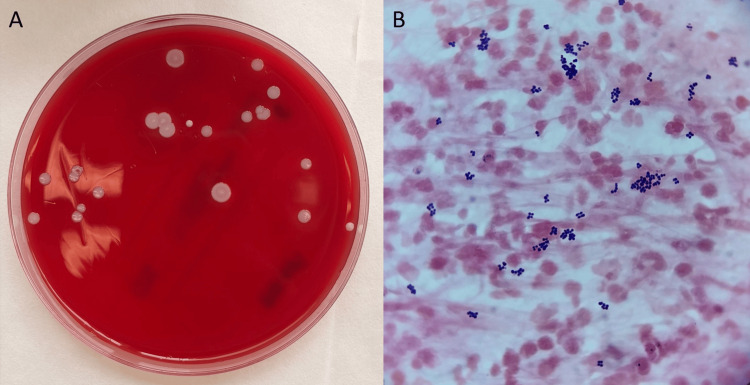
(A) Bacterial culture of Staphylococcus epidermidis on a blood agar plate. White nonhemolytic colonies are observed. (B) Direct microscopic examination of the specimen. Numerous inflammatory cells and Gram-positive cocci grouped in tetrads are observed, consistent with the genus Staphylococcus (Gram stain, 100×)

The presence of white, nonhemolytic colonies on blood agar in bacterial culture indicates *S. epidermidis* (Figure 3A). Direct microscopic examination shows numerous inflammatory cells and Gram-positive cocci grouped in tetrads, indicative of the genus Staphylococcus (Figure 3B).

The patient's postoperative recovery was uneventful, with resolution of the infection. The ophthalmological examination at hospital discharge showed that the left eye had nonreactive mid-dilated mydriasis and amaurosis. The anterior segment was unremarkable, but there was a persistent central preretinal hemorrhage that covered the optic disc. Subsequent investigation confirmed the weapon used was a .25 caliber automatic Colt pistol, designed for short-range shooting, with each bullet weighing 3.24 g.

## Discussion

Gunshot wounds to the head are a frequent cause of morbidity and mortality in both civilian and combat settings. Orbital projectile injuries, in particular, often result in severe damage to the central nervous system and adjacent structures due to their trajectory. A thorough physical examination and initial assessment of multisystem trauma are essential [6].

Initial management should follow the Advanced Trauma Life Support protocol to secure the airway and stabilize the patient. Given that traumatic brain injury is the leading cause of morbidity and mortality, coordinated decision-making with the neurosurgeon is critical. As in this case, the oculoplastic surgeon treats the orbital injuries while the neurosurgeon manages the brain injury. Collaboration between these specialists ensures comprehensive care and reduces complications [2].

According to Viljoen et al. [2] and Fackler et al. [10], projectiles cause tissue damage through several mechanisms: laceration and crushing force along their path; cavitation, which increases with velocity and can destroy adjacent organs; and shock waves, which may produce neurological symptoms. The extent and severity of tissue damage are directly related to the ballistic properties and kinetic energy of the projectile at impact. The kinetic energy of a projectile is determined by its mass and speed; a higher speed results in more energy dispersion and tissue damage. Upon contact with tissues, the bullet's kinetic energy is converted into heat, vibration, mechanical energy, and vacuum effects, all of which contribute to tissue injury [11].

Handguns typically fire projectiles at speeds of around 300 m/s, with bullet weights ranging from 3.2 to 16 g, indicating their potential to cause far more severe injuries than lighter objects such as pellets or metal fragments. Additionally, there is a higher likelihood of damage to adjacent structures such as the sinuses, brain, or orbital walls [6]. As the projectile decelerates within soft tissues of the orbit, energy dissipates [12]. In this case, the bullet became lodged in the retrobulbar space after traversing the frontal sinus and orbital rim.

Sulaiman et al. [4] emphasize that the heterogeneity of injuries in these traumas is influenced not only by the characteristics and velocity of the projectile but also by initial pharmacological treatments. Injuries may range from eyeball alterations to orbital fractures, either minor or complex, involving the floor and other walls, as documented in other studies, which necessitate individualized management of these patients [8,13]. Orbital reconstruction may be required, particularly in cases involving major fractures causing disfigurement or when late-onset enophthalmos develops.

Even in cases where the eye is not directly affected, optic nerve damage or impaired orbital vascularization can cause traumatic optic neuropathy or retinal ischemia, which can lead to vision loss [3]. This could account for the visual impairment seen in the case study.

Ocular motility disorders may also arise from penetrating orbital injuries due to direct damage to the extraocular muscles or tendons caused by laceration, avulsion, or transection. Additionally, muscle or nerve damage can also result from contusions brought on by energy transmission during orbitocranial trauma. Edema, ischemia, bleeding, and the development of hematomas can all worsen this damage [14].

As soon as possible after trauma, a thorough clinical examination should be carried out to rule out globe rupture, detect intraocular foreign bodies, and identify signs of optic neuropathy. Imaging studies are recommended to locate the projectile, assess possible orbital fractures, and evaluate associated injuries in the sinuses or other orbital soft tissues [6]. The most frequent injuries after such traumas, when the globe is spared, are orbital fractures, as seen in our patient, and optic nerve damage, which is an irreversible cause of vision loss, as shown by Elegbede et al. [15].

Early and accurate localization of the projectile facilitates surgical access, reduces operative time, and minimizes tissue damage. Angiography, CT, ultrasound, and plain radiography can all be used to accomplish this [16,17]. CT is the imaging modality of choice for these patients due to its accessibility, rapid acquisition, and high sensitivity in detecting acute hemorrhagic lesions, soft-tissue injuries, and fractures. Additionally, CT scans delineate the trajectory of retained fragments, assist in localization, and aid surgical planning for potential removal [2].

Although the bullet core is typically made of a lead alloy, ferromagnetic materials may still be present. In these cases, magnetic resonance imaging (MRI) is not recommended due to the possibility of projectile displacement, subsequent injury, or heat generation. Consequently, the bullet needs to be removed in advance if an MRI is required [5,16].

The composition of the projectile is also relevant. Typically, an alloy of copper, zinc, or lead, it is generally well tolerated. However, oxidation or prior exposure to plant material can trigger inflammation or infection, factors that influence subsequent management [6]. Although systemic lead poisoning from retained orbital foreign bodies is theoretically possible, no reported cases exist [5].

As noted by several authors [5,9,18], metallic foreign bodies retained in the orbit are generally well tolerated. Only about 5% of cases without globe penetration develop complications such as pain, restricted ocular motility, optic neuropathy, fistula formation, or infection. Less than 10% of inorganic foreign bodies in the orbit cause cellulitis or abscess formation.

The decision to remove the projectile depends on the specific circumstances of each case, weighing risks and benefits. While radiological studies may clearly identify the projectile, intraoperative localization is often challenging because of its deep position within the orbital soft tissues. Surgical manipulation carries risks ranging from orbital hemorrhage to optic neuropathy [18].

If there is little surgical risk or the projectile is palpable, Finkelstein et al. [6], Ho et al. [9], and Fulcher et al. [18] advise removal because it reduces the risk of infection and fistula formation and supports the possibility of future MRI. If the projectile is located posteriorly, initial observation is advised. Extraction should be considered when there is inflammation, infection, orbital abscess, orbital fractures, or radiological evidence of optic nerve compression leading to optic neuropathy, as in our case and in others reported in the literature [3,5].

A projectile lodged near critical structures such as the optic nerve, cranial nerves III, IV, and VI, ethmoidal vessels, or extraocular muscles poses a significant risk. Extreme caution is necessary during removal to avoid injury to these structures [17]. In this case, the patient presented with a large, retrobulbar projectile accompanied by fever, likely secondary to an orbital infection not detected on CT, making removal the most appropriate course of action, along with orbital fracture repair.

Some studies suggest that a brief delay in removal may reduce inflammation, provided other injuries allow it [19]. According to Shuker [17], the projectile should ideally be extracted within 24-48 hours. A fibrous capsule may form around the projectile if removal is delayed, making extraction more difficult because of adhesions with surrounding tissues. This fibrous capsule is called biofilm and is composed of microorganisms attached to a surface and enveloped by an extracellular matrix produced by themselves that prevents the action of the antibiotics.

Initially, we opted for observation given the projectile's location. However, the onset of fever despite intravenous antibiotic treatment raised suspicion of infection associated with the retained bullet, prompting its removal. We emphasize that selecting an appropriate surgical technique, ensuring optimal visualization, and minimizing tissue trauma are critical for successful outcomes, as Finkelstein et al. [6] and Shuker [17] have shown. Careful dissection and meticulous hemostasis further reduce the risk of complications such as hemorrhage or optic nerve damage during surgery.

The lateral orbitotomy approach has proven effective for extracting foreign bodies near the orbital roof or retrobulbar region, while the infraorbital approach is preferred for objects near the orbital floor [17]. In this case, a lateral orbitotomy was performed without removing the orbital rim, which provided access to the deep orbit, allowing successful removal of the bullet. Subsequently, an inferior transconjunctival approach was used to place a titanium mesh on the orbital floor without complications. Following a gunshot wound to the orbit with globe preservation, the most common long-term symptoms are persistent pain and loss of visual acuity [20], as observed in the presented case.

## Conclusions

In conclusion, orbital gunshot injuries are rare and carry a poor prognosis. These injuries require a personalized clinical approach for management. The removal of a bullet lodged in the middle or deep orbit from penetrating trauma presents a significant surgical challenge due to the confined anatomical space and proximity to vital structures. Retained posterior orbital metallic foreign bodies may be observed initially when surgical risk is high and there is no immediate complication. However, development of fever, orbital abscess, optic nerve compression, motility restriction, or other complications should prompt reconsideration of surgical removal. CT localization and multidisciplinary management are essential.
